# Development and psychometric properties of Health-Promoting Lifestyle Scale in Colorectal Cancer Survivors (HPLS-CRCS): a mixed-method study

**DOI:** 10.1186/s12885-023-11436-7

**Published:** 2023-10-03

**Authors:** Elahe Ramezanzade Tabriz, Monir Ramezani, Abbas Heydari, Seyed Amir Aledavood, Jamshid Jamali

**Affiliations:** 1 Department of Medical Surgical Nursing, School of Nursing and Midwifery, Mashhad University of Medical Sciences, Mashhad, Iran; 2 Nursing and Midwifery Care Research Center, Mashhad University of Medical Sciences, Mashhad, Iran; 3 School of Nursing and Midwifery, Mashhad University of Medical Sciences, Ebne Sina St, PO Box 9137913199, Mashhad, Iran; 4 Cancer Research Center, Mashhad University of Medical Sciences, Mashhad, Iran; 5 Department of Biostatistics, School of Health, Mashhad University of Medical Sciences, Mashhad, Iran; 6 Social Determinants of Health Research Center, Mashhad University of Medical Sciences, Mashhad, Iran

**Keywords:** Lifestyle, Health promotion, Colorectal cancer, Survivors, Scale, Psychometrics

## Abstract

**Background:**

Detecting a health-promoting lifestyle in colorectal cancer (CRC) survivors is of paramount importance to manage disease complications, prevent their recurrence, and enhance survival; however, no specialized tool has yet been provided to measure the lifestyle of these patients. Accordingly, this study aimed to develop and determine the psychometric properties of the Health-Promoting Lifestyle Scale in CRC Survivors (HPLS-CRCS).

**Methods:**

This study was a mixed study with an exploratory sequential design in two phases. Concept analysis was performed in the first phase according to Schwartz-Barcott and Kim’s (2000) hybrid model to explain the concept, identify dimensions, and generate items. In the second phase, psychometrics including validity (face, content, and construct) and reliability (internal consistency and stability) were determined. Responsiveness, interpretability, ease of use, item weighting, and scale scoring were also determined.

**Results:**

After explaining the concept, an initial scale encompassing 211 items was developed, content and item analyses were conducted, and the items decreased to 89 items after the face validity assessment. For construct validity, confirmatory factor analysis (CFA) was conducted with a sample size of 500 survivors, and convergent validity was performed for the Persian version of the Health-Promoting Lifestyle Profile II (HPLP-II). Accordingly, 80 items were classified into six factors: activity and rest, spiritual growth, health responsibility, nutrition, interpersonal relationships, and psychological management, with RMSEA = 0.055, χ^2^/df = 2.484, and χ^2^ = 6816.516. The reliability of the scale was confirmed, Cronbach’s alpha was between 0.865 and 0.928, and the intraclass correlation coefficient (ICC), the standard error of measurement (SEM), the minimal important change (MIC), and the smallest detectable change (SDC) were 0.896, 3.36, 13.86, and 19.87, respectively.

**Conclusion:**

The HPLS-CRCS consists of 80 items in six dimensions and is a valid and reliable scale for evaluating the health-promoting lifestyle in CRC survivors. Using this scale to evaluate the healthy lifestyle in these survivors can lead healthcare providers to detect deficiencies and plan the lifestyle of CRC survivors during the post-treatment period.

**Supplementary Information:**

The online version contains supplementary material available at 10.1186/s12885-023-11436-7.

## Background

Colorectal cancer (CRC) is the third most common cancer worldwide [1]. Cancer survival begins with the diagnosis of the disease and continues throughout life. At present, there are about 3.5 million CRC survivors worldwide [2].

In recent years, the financial burden of disease and physical and mental problems in long-term survivors of colorectal cancer has been observed in disease management, stemming from substantial cancer-related time and lifetime out-of-pocket medical expenditures [3]. In the long term, the increased burden of CRC can have negative effects on general health, disease prognosis, and quality of life (QoL) after treatment [4–7]. However, the relationship between the decreased disease burden and mortality rate caused by CRC and a healthy lifestyle is constantly increasing [3, 8]. Evidence indicates that changing unhealthy behaviors and modifying lifestyles among CRC survivors is a potential factor affecting survival positively, reducing recurrence, improving daily functioning, decreasing treatment complications, and improving health-related quality of life (HRQoL) [9–13]. Previous studies have also indicated that unhealthy lifestyles enhance the risk of cardiovascular and metabolic diseases, and that these complications are more likely in CRC patients than in individuals without cancer or individuals with breast and prostate cancer [9]. Moreover, lifestyle changes after CRC diagnosis, including physical activity, proper nutrition, and a body mass index (BMI) ≤ 25, can lower the risk of secondary cancers, treatment complications, risk of recurrence, and mortality caused by other diseases and CRC by 50% [8, 14–16].

Although cancer diagnosis may immediately motivate individuals to adopt a healthier life, awareness of the exact nature of a healthy lifestyle and maintaining it over time after survival is challenging. Furthermore, some of the delayed effects of the disease manifest several years after treatment, when patients may have no motivation to choose a healthy lifestyle and insufficient awareness of how to modify their current lifestyle or may have difficulty implementing it [17]. Accordingly, CRC survivors and healthcare providers should be aware of the extent to which individuals follow a healthy lifestyle.

To evaluate the lifestyle, various general and specialized tools have been designed, which can be mentioned such as the Lifestyle Questionnaire for School-aged Children (LQ), Adolescent Lifestyle Questionnaire (ALQ), Healthy Lifestyle Assessment Questionnaire for the elderly, Mother’s Lifestyle Scale during the pregnancy, Health Promoting Lifestyle Profile II, FANTASTIC Lifestyle Checklist, Weight Efficacy Life-Style Questionnaire (WEL), Healthy Lifestyle Instrument for Breast Cancer Survivors (HLI-BCS), Type 2 Diabetes and Health Promotion Scale (T2DHPS), Lifestyle scale during COVID-19 disease pandemic [17–28]. To review the tools published in the field of lifestyle, it was found that the first tools were designed for the general population, and translation and psychometrics were conducted on a specific population for better use in different cultures and societies. The content of these tools emphasizes the concept of healthy lifestyles and assesses whether people are healthy physically, mentally, and socially [18–20]. With the passage of time and the evolution of lifestyle concepts, specialized tools have been designed based on the developmental and demographic social characteristics of children, adolescents, students, the elderly, and pregnant women [19, 21–23]. Some lifestyle tools are designed based on specific characteristics of chronic diseases such as cardiovascular disease, diabetes, and COVID-19 [25–28]. Hence, a generic tool designed with the concept of a healthy lifestyle in mind cannot be used for CRC survivors, because their lifestyle has changed due to the disease and the consequences of treatment, and evaluation with these tools leads to unreliable results and wastage of time and cost.

To evaluate the health-promoting lifestyle in CRC survivors and determine the status of these individuals in providing better educational and care services, there is a need for an accurate tool exclusive to these survivors. On the other hand, the survivors’ lifestyles can be different from various individuals and groups according to the treatment, care, geographic, and socioeconomic background [8, 9, 14–18]. In this regard, the present study aimed to develop and formulate a standard tool to evaluate this concept over time after contracting cancer. Developing a standard tool to measure CRC survivors’ health-promoting lifestyle allows healthcare providers to have the precise and principled recognition of these patients’ lifestyle status after cancer and treatment and accordingly plan and implement the provision of educational and care services underlining the improvement of CRC survivors’ lifestyles.

## Methods

### Study design and procedures

**Fig. 1:**
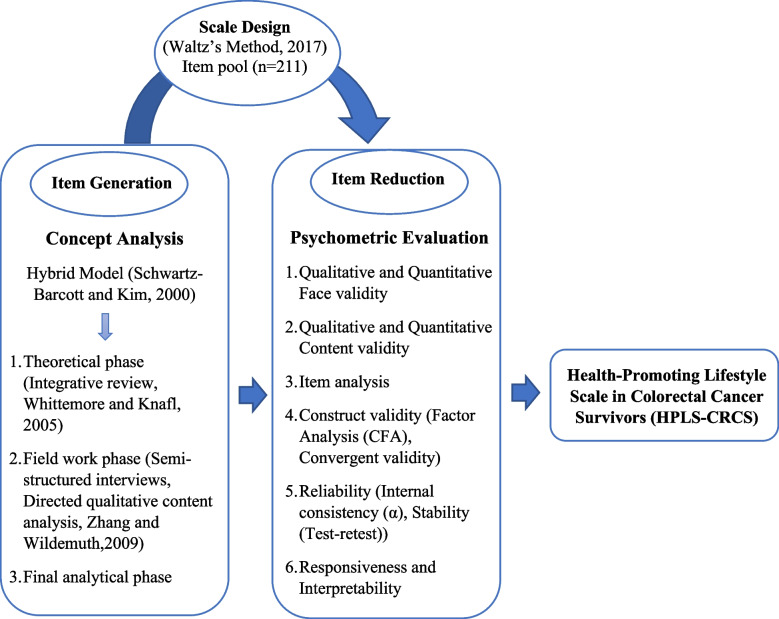
Flow chart of phases of study

The present study was a mixed study with an exploratory sequential design that aimed to develop and assess the psychometric properties of the Health-Promoting Lifestyle Scale in Colorectal Cancer Survivors (HPLS-CRCS) from March 2020 to November 2021. This study consisted of two phases. The first phase included item development using concept analysis and a hybrid method, and the second phase included item reduction according to cross-sectional studies to determine the psychometric properties of the scale (Fig. 1).

### Item generation

Schwartz-Barcott and Kim’s (2000) hybrid model was used in this study to analyze the concept of a “Health-Promoting Lifestyle in CRC Survivors” [29]. This model consists of three phases: (1) Theoretical phase (an integrated review using Whittemore and Knafl’s (2005) approach) [30, 31], (2) Fieldwork phase (collecting the patients and experts’ experiences using semi-structured and in-depth interviews and analyzing Zhang and Wildemuth’s (2009) directed content analysis method) [32, 33], and (3) Final analytical phase (combining the findings of the theoretical and fieldwork phases).

In the theoretical phase, the databases of ProQuest, Medline, Science Direct, Web of Science, Scopus, and national Persian language databases of Magiran and SID were searched for all studies (quantitative, qualitative, and mixed method), books and clinical guidelines published by June 2020. The search keywords were “lifestyle, health promotion, colorectal cancer, colorectal neoplasms, cancer patients, cancer survivors, and lifestyle interventions,” as combined using logical operators (AND/OR). The inclusion criteria were various English or Persian texts or articles published in peer-reviewed journals on CRC survivors’ health-promoting lifestyles. Articles on lifestyle before diagnosis and at the screening CRC stage, journal notes such as short articles of the editorial word type or presenting commentarial ideas, and abstracts of congresses and conferences were excluded from the study.

The articles’ quality was evaluated by two assessors independently using the Mixed Methods Appraisal Tool (MMAT) version 2018, and the Preferred Reporting Items for Systematic Reviews and Meta-Analyses (PRISMA) checklist was also used to assess the review articles [34, 35]. The dimensions of Pender’s Health Promotion Model (HPM) were used for data analysis and reduction [36]. Finally, 167 texts (138 articles, 14 clinical guidelines, and 15 books) were entered into the data extraction stage. Furthermore, the antecedents, attributes, and consequences related to the concept were extracted from the texts, and a comprehensive definition of the concept of a “Health-Promoting Lifestyle in CRC Survivors” was provided based on theoretical phase [37]. Guide and questions for the fieldwork interviews were also prepared at this phase.

In this phase, 45 interviews (12 CRC survivors and 33 experienced healthcare providers) were performed through purposive sampling to detect the participants’ understanding of “Health-Promoting Lifestyle in CRC Survivors”. The interviews were carried out with maximum diversity in selecting participant, face-to-face, in-depth, and semi-structured (20–90 min), from August to December 2020 in clinic or workplace. Each participant was interviewed once by the first author.

Topic guide and interview questions focused on the contradictory and ambiguous findings of the theoretical phase, the components of the health-promoting lifestyle based on HPM [36], the underlying factors of the health-promoting lifestyle in CRC survivors and their experiences.

At the beginning of the interview, the researcher introduced herself and the purpose of the study and ethical considerations, and asked permission from each participant regarding audio recording and written informed consent. After transcribing the recorded data, the interview texts were analyzed using MAXQDA software version 10, using Zhang and Wildemuth’s (2009) directed content analysis method. Data collection and analysis continued until data saturation was attained. It was obtained when no emergence of new subcategories, codes, or data repeated which was expressed in previous interviews [38, 39].

To ensure the reliability of the qualitative data, Guba’s criteria (1981) including credibility, confirmability, dependability, and transferability were considered [39].

In the final analysis phase, the definition of the concept of a “Health-Promoting Lifestyle in CRC Survivors” in the theoretical phase was compared with the experimental findings in the fieldwork phase, resulting in the redefinition of the concept and promotion of the theoretical definition. In this phase, a more complete definition of the combination of the two previous phases was then extracted. The antecedents, attributes, and consequences of this concept were placed in 19 categories and 88 subcategories. According to the analysis of the hybrid concept, “Health-Promoting Lifestyle in CRC Survivors” was defined and explained.

The next stage used the extracted concept definition to design the scale. The initial scale was designed using Waltz’s method (2017) in four stages: selection of a conceptual model, explication of objectives for the measure, development of a blueprint, and construction of the measure [40]. The scale dimensions were determined based on the subcategories and categories of the conceptual analysis. Appropriate items in each dimension were generated based on the extracted codes. The researchers assessed the written structure, difficulty perception, and item similarity, according to which some items were merged or omitted. A set of 211 items was finally prepared and then face validity, content analysis, item analysis and construct validity were performed.

### Item reduction

The psychometric properties of the HPLS-CRCS, including validity (face, content, and construct) and reliability (internal consistency using Cronbach’s alpha coefficient and stability using test–retest), were determined. Finally, responsiveness, interpretability, ease of use, item weighting, and scale scoring were evaluated.

#### Face validity

In qualitative face validity, the level of difficulty, the rate of proportion, and item ambiguity were assessed by 10 CRC survivors, and according to their comments, some items were revised for more clarity in terms of writing. For quantitative face validity, ten other CRC survivors were also asked to specify their comments on the importance of each item based on a five-point Likert scale ranging from “Very Important” (5) to “No Important” (1). The score for each item was calculated using the following formula: impact score = frequency (%) × importance. Items with a score > 1.5 were acceptable, and five items with a score < 1.5 were merged with other similar items, or their writing structure was modified [41–44].

#### Content validity

In qualitative content validity, a panel of experts (*n* = 20) was formed, who were experts in tool design (*n* = 2), nursing faculty members (*n* = 2), radiation oncologist (*n* = 3), oncology nurses (*n* = 3), oncology surgeons (*n* = 2), clinical nutrition specialists (*n* = 2), traditional medicine specialists (*n* = 2), psychiatrists (*n* = 2), a social worker (*n* = 1), and a clergyman (*n* = 1). They were asked to fill out checklists to assess clarity and simplicity, grammar, suitability of words, placement and adequacy of items, and scoring. At this stage, based on the opinions and suggestions of the panel of experts, changes were made on the items. Then the modified items were checked by 20-expert panel in the quantitative content validity stage.

In quantitative content validity, the necessity of including each item (content validity ratio [CVR]) in the scale was determined. According to the table of Scully and Ayre (2014), the acceptable CVR is 0.50 [45]. Then, the relationship between the item and the scale objective (content validity index [CVI]) was assessed for each item and the total scale. CVI > 0.79 is appropriate, if it is 0.70–0.79, the item should be revised, and if it is < 0.70, the item is unacceptable and should be omitted [40]. After considering the opinions and suggestions of the panel of experts and CVI and CVR coefficients, the required modifications were made to the items, and the items were decreased from 211 to 89 items.

#### Item analysis

The item analysis was conducted on 50 CRC survivors before performing the construct validity to assess the correlation between each item and the other items and the scale. At this stage, items with a correlation coefficient of < 0.25 with other items and the scale were omitted [22, 44]. Accordingly, Cronbach’s alpha coefficient was calculated for the items and the scale. No items that lead to a decrease in scale correlation were identified and eliminated, and 89 items entered the construct validity process.

#### Construct validity

In the present study, although data organization in the concept analysis “Health-Promoting Lifestyle in CRC Survivors” was carried out based on the dimensions of Pender’s HPM, the previous assumption of the factors and devoting items to each factor are pre-determined, and construct validity is to confirm or reject this assumption. Accordingly, confirmatory factor analysis (CFA) was performed [46, 47]. The normality of the data was assessed using the data kurtosis and skewness, the Kolmogorov–Smirnov, the Shapiro–Wilk tests, and the Q-Q graph. The test results of 0.271 and 0.762 were obtained at *p* > 0.05, respectively. Since the normality of the data was confirmed, the maximum likelihood method was used to estimate CFA. The fit indices of 1 < χ^2^/df < 3, CFI > 0.9, TLI/NNFI > 0.9, RMSEA < 0.1, and PNFI > 0.5 were used [48]. The Hoelter test was also used to confirm the adequacy of the sample size CFA. Hoelter (1983) introduced the Critical N (CN) statistic for the evaluation of SEM sample size, where CN ≥ 200 was considered adequate. After data collection and SEM model specification, we could estimate the post-hoc sample power with the non-centrality parameter (NCP or λ). Sample size N equals (NCP/Fmin) + g. Hence, it could obtain the Fmin value from the model, calculate the NCP for a given df, critical chi-square, and power then calculate the sample size (N) using these values [49, 50]. According to this test, with an error rate of 0.05, the minimum sample size for CFA in this study was sufficient to be 210.

In this study, this cross-sectional descriptive study used the convenience sampling method to select 500 CRC survivors meeting the inclusion criteria for construct validity. Data collection was conducted in person or online by sending a form via virtual platforms.

#### Convergent validity

To determine convergent validity, 50 CRC survivors were asked to complete the Persian version of the Health Promoting Lifestyle Profile II (HPLP-II) and the HPLS-CRCS simultaneously [18], and the correlation between the two scales was determined. The Pearson’s correlation coefficient ≥ 0.40 was used for the convergent validity.

#### Reliability

The internal consistency and stability of the scale were tested to ensure reliability. To assess the internal consistency, 30 CRC survivors filled out the scale, and Cronbach’s alphas of the total scale and factors were then calculated. Cronbach’s alpha values range from 0 to 1, and a minimum value of 0.7 is recommended [51]. Higher values indicate acceptable reliability. To determine stability over time, the intraclass correlation coefficient (ICC) was calculated. To this end, 20 CRC survivors completed the final scale in two periods with an interval of two weeks. The ICC value ranges from 0 to 1, the reliability coefficient of 0.6 is acceptable, and the reliability ≥ 0.8 indicates excellent stability. Regarding the stability of the scale, the standard error of measurement (SEM) was also calculated using SD × √ (1-ICC agreement) = SEM agreement [41].

#### Responsiveness and interpretability

Responsiveness refers to the assessment of changes over time. According to the consensus-based standards for the selection of health measurement instruments (COSMIN), the SEM comparison, the minimal important change (MIC), and the smallest detectable change (SDC) are used to determine the response rate of the scale [52, 53]. Responsiveness is confirmed if SDC > MIC, MIC < 30%, and MIC < 10% are considered acceptable and excellent, respectively. Interpretability refers to the ability to attribute qualitative meaning to quantitative scores. Regarding the interpretability of a scale, the percentage of missing data, data distribution, and MIC and SDC indices should be calculated, and floor and ceiling effects exist if above 20% of respondents obtain the minimum or maximum scores, respectively [54].

#### Multivariate normality and outliers

The univariate and multivariate distributions were assessed in terms of kurtosis ± 7, skewness ± 3, and Mardia’s coefficient > 8 for outliers, respectively. Multivariate outliers were determined by evaluating items with Mahalanobis distance (*p* < 0.001) [41, 47]. All statistical analyses were performed using IBM SPSS software version 24 and IBM SPSS AMOS software version 25. In this study, *p* < 0.05 was considered statistically significant.

## Results

### Item development

After comparing and combining the definitions of the two theoretical and field work phases, the final operational definition of the concept of “Health-Promoting Lifestyle in CRC Survivors” was provided. This concept consisted of six attributes (health responsibility, nutrition, activity and rest, interpersonal relationships, spiritual growth, and psychological management), six antecedents (demographic, clinical, physical, psychological, and socioeconomic variables, and time and environmental restrictions), and seven consequences (disease prognosis improvement, physical status improvement, psychological status improvement, social status improvement, QoL improvement, economic well-being improvement, and public health improvement). Based on the attributes defined for the concept, 211 initial items were extracted.

### Item reduction

#### Face and content validity

The clarity, appropriateness, and impact score of each item were assessed in face validity, and 206 items assessed for content validity. According to the comments of the 20-expert panel and CVR, 62 items with scores < 0.50 were omitted. Three items with scores < 0.70 were omitted regarding the CVI score at this stage. Moreover, 126 items with scores between 0.70 and 0.79 were revised by the researchers or merged with other items. S-CVI/Ave was 0.91. Finally, 89 items entered the construct validity.

#### Construct validity

**Fig. 2:**
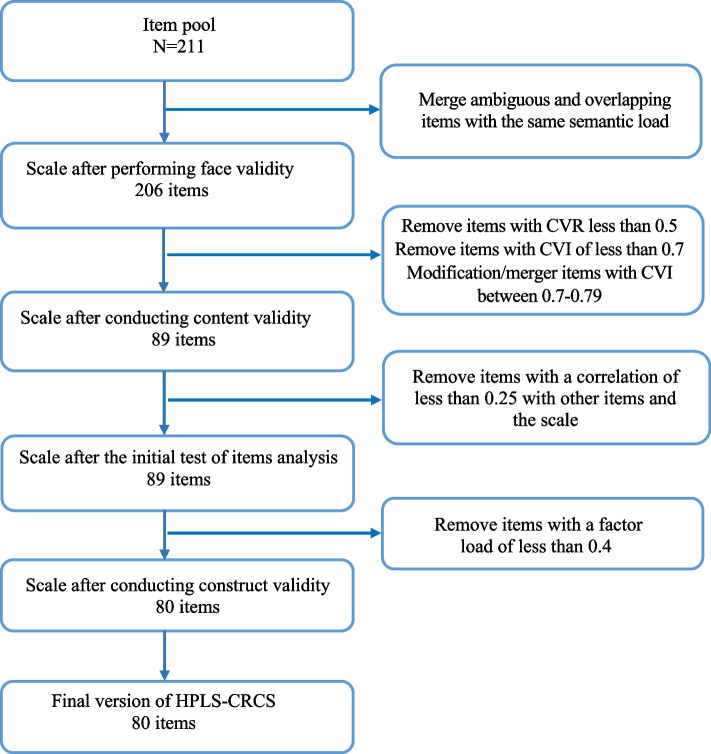
Flow chart of item reduction HPLS-CRC items

**Fig. 3:**
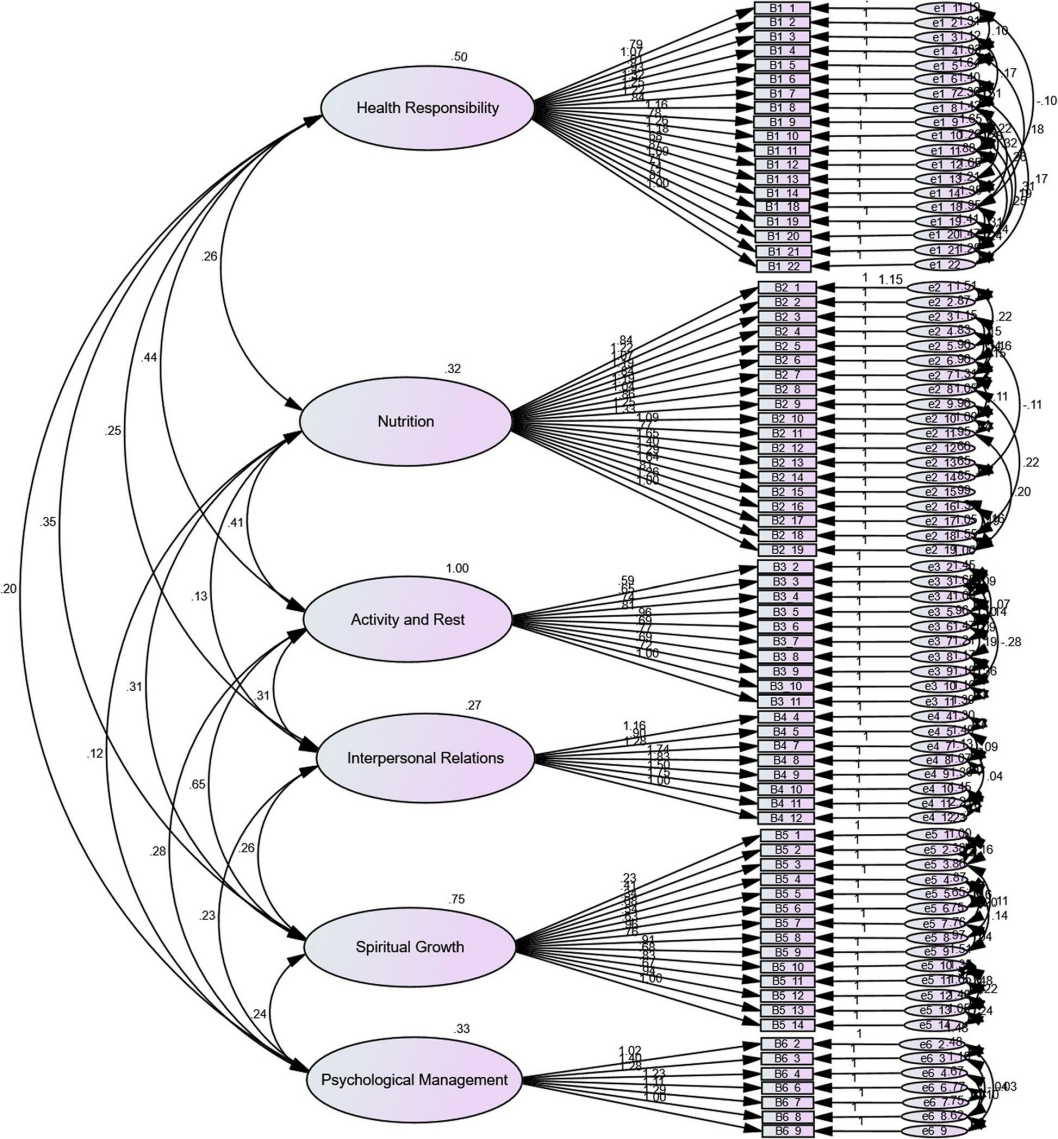
The HPLS-CRCS construct: modified model of second-order confirmatory factor analysis (*N* = 500)

Five hundred CRC survivors with a mean age and standard deviation of 56.6 ± 6.1 years and a history of disease contraction of 4.7 ± 0.4 years participated in construct validity (Table 1). After drawing the preliminary conceptual model and calculating the fit indices, we had 3 < χ^2^/df, CFI and TLI/NNFI were not < 0.9, and RMSEA < 0.1. Accordingly, in the first-order CFA, the data analysis and outlier identification were carried out for model modification, during which no outlier was identified; however, nine items with factor loadings of < 0.4 were removed (Fig. 2). Then, the inter-error covariance was drawn in the second-order CFA based on the model’s modification indices. The clinically justifiable and valuable parameters of a factor were assessed, and correlations were determined between the errors with valuable covariance, which could further affect the fit indices (Fig. 3). The model was finally confirmed with acceptable fit indices and 80 items (Table 2).

**Table 1:** Socio-demographic and clinical information of participants in construct validity (*N* = 500)

**Variables**		**n (%)**
**Gender**	Female	239(47.8)
	Male	261(52.2)
**Age (Year)**	30–18	24(4.8)
	50–31	166(33.4)
	70–51	309(61.8)
**Marital status**	Single	23(4.6)
	Married	393(78.6)
	Divorced	14(2.8)
	Widow	70(14)
**Education status**	Under diploma	269(53.8)
	Diploma	139(27.8)
	Academic	92(18.4)
**Occupational status**	Unemployed	225(45)
	Employed	129(25.8)
	Retired	146(29.2)
**Economic status**	Poor	286(57.2)
	Moderate	210(42)
	Good	4(0.8)
**Time since diagnosis (Year)**	> 5	414(82.8)
	< 5	86(17.2)
**Treatment type**	Surgery	9(1.8)
	Surgery and Chemotherapy	176(35.2)
	Surgery and Radiotherapy	90(18)
	Combination of treatments	225(45)
**Stage of disease**	I	101(20.2)
	II	215(43)
	III	89(17.8)
	IV	95(19)
**History of Comorbidity**	Yes	398(79.6)
	No	102(20.4)

**Table 2:** Fitness indices in the first and second-order factor analysis (*N* = 500)

**Indexes**	**Cut-off values**	**First-order**	**Second-order**
χ^2^ /df	< 3	2.241	2.484
*P-* value	≥ 0.05	< .0001	< .0001
RMSEA (Root Mean Square Error of Approximation)	0.05 < RMSEA < 0.08	0.061	0.055
PNFI (Parsimonious Normed Fit Index)	PNFI > 0.5	0.613	0.627
CFI (Comparative Fit Index)	0.90 < CFI	0.757	0.930
GFI (Goodness of fit index)	0.90 < GFI	0.758	0.899
TLI/NNFI (Tucker–Lewis index /Non normed fit index)	0.09 < TLI/NNFI	0.875	0.909

For convergent validity, the correlation between the scores of the two Persian version tools of HPLP-II and HPLS-CRCS were calculated, and the correlation coefficient of 0.761 was obtained at *p* < 0.001, which is favorable considering Pearson’s correlation coefficient of ≥ 0.40.

#### Reliability, responsiveness, and interpretability

The reliability of the scale was acceptable, Cronbach’s alpha for each category ranged from 0.865 to 0.928, and the ICC value for the total scale was 0.869 and 0.645–0.962 with a 95% confidence interval, indicating the stability of the scale over time. To assess responsiveness, the SEM value for the total scale was 3.36, suggesting that an estimate of the expected deviation rate in a group of measurements in a specific situation of the values is real. The MIC value reveals 13.86 for the total scale. Also, the SDC value for the whole scale was 19.87, which is a larger value than the MIC value, indicates agreement in the scale. Calculating ceiling and floor effects revealed that the minimum and maximum scores for all dimensions and the total scale were < 20% (Table 3).

**Table 3:** Internal consistency, construct reliability and descriptive statistics, floor and ceiling effects of HPLS-CRCS

**Factor**	**No. items**	**Mean (SD)**	**Alpha (CI95%)**	**ICC (CI95%)**	**SEM**	**SDC**	**MIC**	**Floor effect (%)**	**Ceiling effect (%)**
Activity and Rest	10	3.05(0.87)	0.839(0.818–0.859)	0.729(0.664–0.862)	3.18	7.09	3.80	0	0.8
Spiritual Growth	14	3.75(0.68)	0.853(0.893–0.952)	0.786(0.516–0.910)	2.18	6.23	4.46	0	3
Health Responsibility	19	3.05(0.75)	0.861(0.843–0.878)	0.961(0.864–0.989)	3.44	8.43	4.23	0	0.6
Nutrition	19	3.54(0.69)	0.886(0.891–0.937)	0.820(0.597–0.948)	3.89	10.24	6.75	2.5	0
Interpersonal Relations	10	2.98(0.81)	0.773(0.742–0.821)	0.918(0.714–0.976)	1.73	3.12	1.29	0.2	0.8
Psychological Management	8	2.25(0.70)	0.798(0.770–0.823)	0.879(0.552–0.946)	1.43	3.91	2.22	0	0
HPLS-CRCS	80	3.20(0.58)	0.895(0.865–0.928)	0.896(0.645–0.962)	3.36	19.87	13.86	0	0

#### Ease of use

In determining reliability and stability, the mean response time of 15 CRC survivors was calculated. The mean time to complete the scale was 23 min (range 20–26). Furthermore, the participants responded to all items to calculate the percentage of non-responsiveness for each item in construct validity. To make the scale easier to complete, the instruction was initially provided as a guide.

#### Scoring items

This questionnaire was scored on a five-point Likert scale in the form of “never, rarely, sometimes, often, and always,” with scores ranging from 1 to 5 (never (1) and always (5)). In each subscale, the conversion of the linear formula was used in two ways without item weight (all weights = 1) and the scoring item weight for a better understanding of scoring and comparability of scores. The linear formula is as follows:

$$\mathrm{Score\ by\ percentage }= \frac{\mathrm{Raw\ score\ obtained}-\mathrm{ Minimum\ possible\ raw\ score\ possible}}{\mathrm{Maximum\ possible\ raw\ score }-\mathrm{ Minimum\ possible\ raw\ score }} \times 100$$

Finally, the minimum and maximum possible scores range from 80 to 400 without the item weight and from 96 to 480 regarding the item weight. A higher mean score denotes an optimal health-promoting lifestyle in CRC survivors.

## Discussion

The HPLS-CRCS consists of 80 items in six dimensions (activity and rest, spiritual growth, health responsibility, nutrition, interpersonal relationships, and psychological management) and is a valid and reliable scale to evaluate the health-promoting lifestyle in CRC survivors. Since the complications of the disease and treatment in CRC, including chemotherapy and radiotherapy-related problems, changes in excretion habits and control of urine and feces, and disturbances in various body systems, distinguish the lifestyle of these individuals from others [55]. There was no special tool designed for these survivors. What distinguishes this scale from other lifestyle tools is its specificity in measuring lifestyle in the post-treatment period in CRC survivors.

In the present study, the concept analysis based on the hybrid model was used to form the item pool and explain the concept of the HPLS-CRC. On the contrary, item generation approaches are different in lifestyle scales. The majority of them are item generation based on the existing documents and other tools [21, 26, 28], and a limited number of tools, such as the Elderly Lifestyle Questionnaire, the FANTASTIC Questionnaire, and the Adolescent Lifestyle Questionnaire (ALQ), have been designed based on interviews with the target group or Delphi and focus group sessions [20, 22, 23]. In the concept analysis of the hybrid model, the patients and specialists’ existing knowledge and experiences are comprehensively collected, thereby providing a more complete perception and creating a vivid picture of the concept with an emphasis on its clinical aspects [29].

The HPLS-CRCS items cover the specialized aspects of the health-promoting lifestyle in CRC survivors, and the validity and reliability of the scale have also been evaluated using different methods, according to which favorable and acceptable results for the psychometrics have been obtained. After reviewing other lifestyle tools, such as the HPLP-II, the Mothers Lifestyle Scale During Pregnancy, the Type 2 Diabetes Health-Promotion Scale (T2DHPS), Lifestyle Scale During COVID-19 Disease Pandemic, and the Healthy Lifestyle Instrument for Breast cancer (HLI-BCS), various validity and reliability methods have been used for psychometrics. In this study, the indices were mentioned at a favorable level, and their psychometrics was confirmed [18, 19, 25, 27, 28].

For the present scale, responsiveness and interpretability were calculated using the SEM, MIC, and SDC for each factor, and the total scale and ceiling and floor effects were also determined. The results were acceptable and at a favorable level for the HPLS-CRCS. None of the items mentioned in other lifestyle tools has been calculated and reported [18–28], while according to COSMIN evaluation criteria (2010, 2018), the necessity of calculating SEM, MIC, and SDC has been emphasized for the interpretability and responsiveness of health-related patient-reported outcome measures (HR-PROMs) [56].

To evaluate the responsiveness of the scale, an average time of 23 min was set for 80 items. In this regard, given that the shortening of the tool affects the scale’s ease of use and reliability [57, 58], the researchers planned to develop a short-form version in a future study so that less time is spent achieving accurate responses on the physical, psychological, and social conditions of CRC survivors.

According to the CFA results, six factors, including activity and rest, spiritual growth, health responsibility, nutrition, interpersonal relationships, and psychological management, were confirmed for the HPLS-CRCS. The dimension ‘activity and rest’ was first in terms of item factor loadings in CFA, weighting, and item scoring, the subscales of which were measuring regular sleep, favorable sleep quality, reducing sedentary behaviors, having desired sports activity in terms of type, intensity, quality, and duration. This dimension exists in all other lifestyle tools, showing its importance [18–28] since the evaluation and implementation of physical activity improvement programs can positively affect different parts of individuals’ QoL. Evidence reveals that the role of physical activity in reducing the risk of premature death, managing chronic health problems, improving heart health and respiration, reducing the risks of overweight and obesity, maintaining healthy bones, joints, and muscles, reducing the risk of depression, and reducing the risk of CRC occurrence and recurrence cannot be disregarded [59, 60].

The second dimension, spiritual growth, deals with the individual’s ability to grow internally, discover, and express the main goal of life. This dimension measures the individual’s religious and spiritual beliefs and the use of spiritual therapy solutions. The evaluation of spiritual growth is a comprehensive and crucial approach in the field of health because spiritual beliefs affect each person’s interpretations of life events and his/her health status. Understanding patients’ spiritual needs better enables healthcare providers to apply appropriate and influential spiritual interventions tailored to each individual’s condition and status [36, 61]. Moreover, improving the resilience power with different spiritual therapy approaches can affect psychological well-being, improve QoL, reduce depression, and decrease negative emotions in CRC patients [62, 63]. Spiritual beliefs and behaviors in cancer patients are also rooted in the sociocultural context of society; hence, their precise recognition can help oncology nurses and other healthcare providers in developing and implementing better care plans [64].

Health responsibility measures the levels of treatment adherence and self-management of the disease. This dimension deals with acceptance, follow-up, and responsiveness for activities that can direct individuals toward health and make healthy behaviors persistent. The dimension aims to evaluate individuals’ perception of follow-up activities for maintaining health and also the patient’s ability to compare his/her experiences with other useful experiences during health and illness, according to which each person can choose a healthy and favorable behavioral pattern and then implement it [36]. In the majority of patients’ lifestyle questionnaires, this dimension has been introduced as one of the principal dimensions with a high variance percentage [25–28].

The fourth dimension is the nutrition dimension, with considerations addressing nutrients’ preparation and processing, a healthy diet pattern, and the food pyramid, particularly the consumption of bread, grains, fruits, and vegetables. Evaluating the nutritional status and eating habits is considered a crucial part of the comprehensive assessment of health status in individuals, families, and special groups. The data evaluation and analysis specify that planning and implementing which interventions are most appropriate to improve nutritional status, particularly in individuals with nutritional problems such as CRC survivors. Encouraging appropriate nutrition is one of the main concerns in health prevention and promotion and important facets of self-care, which is of paramount importance in improving CRC survivors’ lifestyles [65–67].

Another dimension of the scale is interpersonal relationships, which highlights receiving support from family, friends, healthcare providers, and the workplace. Positive interpersonal relationships support individual efforts and changes to modify lifestyle. For example, attendance in social networks, attendance in society as an active member, and family and friends’ encouragement help individuals stabilize their behavioral changes toward a healthy lifestyle over the long term. The likelihood of successful behavior change is enhanced by promoting the awareness of an individual and his/her supporters and individual and group planning in the family and society [36]. Thus, evaluating and identifying patient support systems and improving interpersonal relationships are effective in achieving comprehensive care. Developing and evaluating nursing interventions to enhance receiving support and improve interpersonal and social relationships at home and in the family, among friends, in the workplace, and in virtual environments via modern-of-the-art technologies promotes individual and social well-being [68].

The dimension of psychological management in the present scale deals with all psychological problems of CRC survivors, including fear of recurrence, depression, lack of adaptation to the disease, post-traumatic stress disorder (PTSD), fatigue, disappointment, social isolation, and even having a recreation and entertainment program to improve psychological disorders. Unlike similar questionnaires exclusively addressing patient stress management [20, 25, 28], this dimension is much more comprehensive. Despite numerous psychological disorders, precise evaluation in these patients is of fundamental importance to enhance the survival rate and longevity for prevention and access to influential and helpful psychological treatments. Regarding psychological interventions, by identifying these problems, oncology nurses can play a crucial role in providing better help to patients in coping with the disease and its related suffering and discomfort and finding life’s meaning under this special condition [68, 69].

### Limitations

Due to the outbreak of the coronavirus disease 2019 (COVID-19) outbreak, it was not possible to include a large number of survivors in the construct validity assessment phase. Therefore, the online scale was sent to and completed by eligible individuals. However, the lack of face-to-face contact with the participants may be one of this phase’s limitations.

### Implications

Using the HPLS-CRCS allows healthcare providers to achieve a precise and fundamental perception of CRC survivors’ post-treatment lifestyle. Identifying the impairments in these survivors’ lifestyles can effectively help plan more appropriate and precise care to reduce the disease recurrence, decrease the economic burden on families and the treatment system, enhance the individuals’ survival, and improve their QoL.

## Conclusion

The findings of this study indicate that the HPLS-CRCS, which is introduced for the first time, is a valid and reliable scale to evaluate the lifestyle of CRC survivors. This 80-item self-report scale can be used for all CRC survivors in the post-treatment phase, who can communicate verbally and in writing. This scale and its results can underlie the better planning and implementation of medical, care, educational, counseling, and support services for healthcare providers, particularly nurses, thereby leading to life pattern changes via health-promoting behaviors and lifestyle improvement.

### Supplementary Information

**Additional file 1.** Health-Promoting Lifestyle Scale in Colorectal Cancer Survivors (HPLS-CRCS).

## Data Availability

The datasets used and/or analyzed during the current study are available from the corresponding author upon reasonable request.

## Abbreviations

CRC: Colorectal cancer
HPLS-CRCS: Health-Promoting Lifestyle Scale in CRC Survivors
HPLP-II: Health-Promoting Lifestyle Profile II
ICC: Intra-Class Correlation coefficient
CFA: Confirmatory Factor Analysis
SEM: Standard Error of Measurement
MIC: Minimal Important Change
SDC: Smallest Detectable Change
QoL: Quality of Life
HRQoL: Health-Related Quality of Life
BMI: Body Mass Index
MMAT: Mixed Methods Appraisal Tool
PRISMA: Preferred Reporting Items for Systematic Reviews and Meta-Analyses
HPM: Health Promotion Model
CVR: Content Validity Ratio
CVI: Content Validity Index
RMSEA: Root Mean Square Error of Approximation
CFI: Comparative Fit Index
GFI: Goodness of Fit Index
PNFI: Parsimonious Normed Fit Index
TLI/NNFI: Tucker–Lewis Index /Non-Normed Fit Index
CN: Critical N
NCP: Non-centrality parameter
SEM: Structural Equation Modeling
COSMIN: COnsensus-based Standards for the selection of health Measurement Instruments
LQ: Lifestyle Questionnaire for School-aged Children
WEL: Weight Efficacy Life-Style Questionnaire
ALQ: Adolescent Lifestyle Questionnaire
T2DHPS: Type 2 Diabetes Health-Promotion Scale
HLI-BCS: Healthy Lifestyle Instrument for Breast cancer
HR-PROMs: Health-Related Patient-Reported Outcome Measures
PTSD: Post-Traumatic Stress Disorder
