# Supplementary material for: Development and psychometric properties of Health-Promoting Lifestyle Scale in Colorectal Cancer Survivors (HPLS-CRCS): a mixed-method study
Source: BMC Cancer. 2023 Oct 3;23:931. doi: 10.1186/s12885-023-11436-7 (PMC10546727; doi:10.1186/s12885-023-11436-7)
Supplement: Supplementary file 1 — Additional file 1. Health-Promoting Lifestyle Scale in Colorectal Cancer Survivors (HPLS-CRCS). [file 12885_2023_11436_MOESM1_ESM.docx]

| **Health-Promoting Lifestyle Scale in Colorectal Cancer Survivors (HPLS-CRCS)** | | | | | | | |
| --- | --- | --- | --- | --- | --- | --- | --- |
| **Dimensions** | **No** | **Items** | **Never** | **Rarely** | **Sometimes** | **Often** | **Always** |
| Activity and rest | 1 | I limit sedentary activities (e.g., long-term drives, watching TV, and lying down during waking hours). |  |  |  |  |  |
|  | 2 | In my usual daily activities, I do sports such as going up and down the stairs instead of using the elevator and parking the car far from the destination, and walking to the workplace. |  |  |  |  |  |
|  | 3 | I do physical activities with mild to moderate intensity at least five days a week for 30 minutes. |  |  |  |  |  |
|  | 4 | I use regular rest periods to prevent physical problems and fatigue during exercise and work. |  |  |  |  |  |
|  | 5 | I regularly wake up in the morning and sleep two hours after dinner. |  |  |  |  |  |
|  | 6 | I use an appropriate place for sleep and relaxation exercises such as yoga, deep breathing, and meditation. |  |  |  |  |  |
|  | 7 | I limit sleep-disturbing activities to a few hours before bed (e.g., exercising, eating heavy foods, using electronic devices, smoking, and consuming alcohol). |  |  |  |  |  |
|  | 8 | I contribute to doing household chores (e.g., cleaning, vacuuming, cooking, home maintenance, and gardening). |  |  |  |  |  |
|  | 9 | I do aerobic, stretching-muscular, and recreational sports (e.g., jogging, cycling, swimming, gardening, aerobics, volleyball, and simple basketball) during the week, according to my physical conditions and physical weakness. |  |  |  |  |  |
|  | 10 | I try to reach an optimal heart rate during sports activities. |  |  |  |  |  |
| Spiritual growth | 11 | I feel satisfaction and peace in life. |  |  |  |  |  |
|  | 12 | I accept changes made in the meaning of life due to suffering from diseases. |  |  |  |  |  |
|  | 13 | I plan to achieve my goals in life. |  |  |  |  |  |
|  | 14 | I devote a quiet place to myself for worshiping God. |  |  |  |  |  |
|  | 15 | If possible, I travel to recreational and religious places. |  |  |  |  |  |
|  | 16 | Despite my trust in God, I did not give up treatment. |  |  |  |  |  |
|  | 17 | I believe in a superior being, like God, and I ask Him for help in difficulties. |  |  |  |  |  |
|  | 18 | With the onset of illness, I care more about myself and my relationship with God. |  |  |  |  |  |
|  | 19 | I improve my attitude toward death as eternal life. |  |  |  |  |  |
|  | 20 | I use methods to reinforce spiritual health, e.g., strengthening relationships with family, society, and friends, attending group prayer meetings, and attending spiritual empowerment courses. |  |  |  |  |  |
|  | 21 | I use religious and literary sources, including books, audio, and spiritual motivational videos. |  |  |  |  |  |
|  | 22 | I use the lives of religious leaders and social heroes as models. |  |  |  |  |  |
|  | 23 | I talk with spiritual individuals about issues obsessing my mind (e.g., God’s wisdom, the disease acceptance, values, beliefs, and meaning of individual life, and worry about death and guilt). |  |  |  |  |  |
|  | 24 | I participate in group sessions aiming at sharing the spiritual sense and experiences with individuals having similar diseases. |  |  |  |  |  |
| Health responsibility | 25 | I carry out treatment follow-ups (e.g., examination, blood test, CT scan, and colonoscopy) as recommended by the physician. |  |  |  |  |  |
|  | 26 | I refer to a specialist to control the abnormal complications of the disease and prevent the contraction of other diseases. |  |  |  |  |  |
|  | 27 | I avoid excessive and arbitrary use of drugs, painkillers, and medicinal supplements. |  |  |  |  |  |
|  | 28 | When necessary, I do drug therapy and appropriate exercises and make certain foods to improve digestive problems, including diarrhea or chronic constipation. |  |  |  |  |  |
|  | 29 | Referring to a urologist, I follow up on diet modification, drug therapy, and adherence to treatment plans. |  |  |  |  |  |
|  | 30 | In case of sexual problems after the treatment, I would start the treatment by referring to a specialist physician. |  |  |  |  |  |
|  | 31 | In the case of chronic pain, by referring to pain specialists to receive rehabilitation and palliative care, I use treatment methods. |  |  |  |  |  |
|  | 32 | I do the vaccination after consultation with a specialist physician, if necessary. |  |  |  |  |  |
|  | 33 | To prevent skin injuries, I use protective methods such as wearing sunscreen, hats, sunglasses, and appropriate clothing. |  |  |  |  |  |
|  | 34 | In oral and dental care, I do regular and daily brushing, use dental floss and mouthwash containing fluoride, and do periodical examinations. |  |  |  |  |  |
|  | 35 | I use educational materials and programs for disease management, treatment, and required changes in lifestyle. |  |  |  |  |  |
|  | 36 | Regarding proper self-care, I request necessary information from healthcare providers (physician /nurses). |  |  |  |  |  |
|  | 37 | I consume traditional medicine after consultation with the treating physician and under the supervision of a traditional medicine specialist. |  |  |  |  |  |
|  | 38 | If necessary, I consume complementary medicine methods such as acupuncture, massage therapy, homeopathy, energy therapy, and yoga under the supervision of the physician. |  |  |  |  |  |
|  | 39 | I follow the gradual withdrawal of tobacco, including cigarettes, hookah, and narcotics, under the supervision of a specialist physician and a psychologist with drug therapy and behavioral therapy. |  |  |  |  |  |
|  | 40 | I have no weight change above one kilogram per week. |  |  |  |  |  |
|  | 41 | I maintain my weight within the normal range. |  |  |  |  |  |
|  | 42 | I calculate the calories of each meal and its volume and change it based on my weight. |  |  |  |  |  |
|  | 43 | With the guidance of a specialist or a nutritionist, I follow up on the required changes in my diet. |  |  |  |  |  |
| Nutrition | 44 | I learn and apply the right methods of cooking healthy food and washing fruits and vegetables. |  |  |  |  |  |
|  | 45 | When buying ready-made food, I pay attention to its label and nutritional value. |  |  |  |  |  |
|  | 46 | I have regular meals. |  |  |  |  |  |
|  | 47 | I limit the consumption of spicy, salty, sour, fried, grilled, and smoked foods. |  |  |  |  |  |
|  | 48 | I use antioxidant substances, including turmeric, garlic, cinnamon, saffron, and cumin, along with meals. |  |  |  |  |  |
|  | 49 | I observe the restriction of liquid consumption during or immediately after food. |  |  |  |  |  |
|  | 50 | I observe the restriction on the consumption of flatulent foods. |  |  |  |  |  |
|  | 51 | I consume laxative foods more. |  |  |  |  |  |
|  | 52 | I consume chicken, poultry, and fish (at least seven times per month or 100 grams per week). |  |  |  |  |  |
|  | 53 | I consume legumes, nuts, and kernels 3-4 times a week. |  |  |  |  |  |
|  | 54 | I limit the consumption of red meat and high-fat meat products (consumption of <100 grams of red meat or <50 grams of meat products). |  |  |  |  |  |
|  | 55 | I use unrefined whole grains and sprouts, including bread, rice, pasta, and beans. |  |  |  |  |  |
|  | 56 | I consume fruits and vegetables at least five units a day. |  |  |  |  |  |
|  | 57 | I use raw fruits and vegetables containing dark green, yellow, and orange pigments (e.g., tangerines, persimmons, bananas, pears, apples, spinach, lettuce, celery, tomatoes, carrots, cucumbers, and all kinds of onions and garlic). |  |  |  |  |  |
|  | 58 | I use dairy products and low-fat/fat-free probiotic products. |  |  |  |  |  |
|  | 59 | I use vegetable oils, including olive oil, canola oil, fish oil, and oilseeds such as sesame and corn, instead of using trans and saturated fats. |  |  |  |  |  |
|  | 60 | I restrict the consumption of sugar and salt added to food. |  |  |  |  |  |
|  | 61 | I drink more liquids and less tea during the day. |  |  |  |  |  |
|  | 62 | I take multivitamins and mineral supplements (e.g., vitamins E, D, C, A, folic acid, magnesium, zinc, iron, and calcium) by checking the tests under the specialist physician’s supervision. |  |  |  |  |  |
| Interpersonal relationships | 63 | I accept support from my wife, family, and friends while maintaining my independence. |  |  |  |  |  |
|  | 64 | I talk about my problems and concerns with those around me. |  |  |  |  |  |
|  | 65 | I ask for advice or guidance from healthcare providers (doctors/nurses) to follow up healthcare. |  |  |  |  |  |
|  | 66 | I get the answers to my questions precisely and patiently from the healthcare providers (physician /nurses). |  |  |  |  |  |
|  | 67 | After treatment, I start activities and communications with the social environment as soon as possible. |  |  |  |  |  |
|  | 68 | To start working again, I will reinforce my personal abilities and skills by participating in training courses. |  |  |  |  |  |
|  | 69 | To assess the readiness to start work, I refer to a specialist physician and a psychologist or a psychiatrist. |  |  |  |  |  |
|  | 70 | At the workplace, I ask the employer for a flexible work schedule and reduced daily working hours to facilitate my tasks. |  |  |  |  |  |
|  | 71 | I create job security in the workplace by having job insurance, health insurance, and receiving emotional support from co-workers. |  |  |  |  |  |
|  | 72 | I do not do heavy tasks and duties (e.g., lifting heavy objects, sitting and standing for a long time, and bending and kneeling suddenly) at the workplace. |  |  |  |  |  |
| Psychological management | 73 | I am well-prepared to experience and adapt to a new life. |  |  |  |  |  |
|  | 74 | I participate in specialized classes and courses to promote my self-awareness and strengthen my cultural-artistic capabilities. |  |  |  |  |  |
|  | 75 | Referring to a psychologist or psychiatrist, I follow up on the treatment for stress caused by the disease diagnosis and the fear of its recurrence. |  |  |  |  |  |
|  | 76 | I participate in programs to know fellow patients and successful survivors in various scientific, sports, and occupational fields. |  |  |  |  |  |
|  | 77 | I participate in group therapy and provide guidance to fellows who are at the onset of diagnosis. |  |  |  |  |  |
|  | 78 | To improve the relationship with those around my wife and me, I refer to a psychologist, psychiatrist, social worker, and an experienced clinical physician. |  |  |  |  |  |
|  | 79 | Under the supervision of a psychiatrist or psychologist, I use non-pharmacological treatments (e.g., occupational therapy, group therapy, cognitive therapy, awareness therapy, behavioral therapy, relaxation exercises, and yoga) to decrease psychological disorders. |  |  |  |  |  |
|  | 80 | I have lateral recreational activities based on my interests (e.g., reading books and newspapers and using movies and music). |  |  |  |  |  |
